# A Retrospective Case–Control Study on the Differences in the Effectiveness of Theta-Burst Stimulation Therapy for Depression with and without Antidepressant Medication

**DOI:** 10.3390/jcm13020399

**Published:** 2024-01-11

**Authors:** Haruki Ikawa, Yuya Takeda, Ryota Osawa, Akiko Sato, Hoshimi Mizuno, Yoshihiro Noda

**Affiliations:** 1 Tokyo Yokohama TMS Clinic, Kawasaki 211-0063, Japan; 2 Department of Neuropsychiatry, Keio University School of Medicine, Tokyo 160-8582, Japan

**Keywords:** major depressive disorder (MDD), treatment-resistant depression (TRD), intermittent theta-burst stimulation (iTBS), transcranial magnetic stimulation (TMS), antidepressants

## Abstract

Transcranial magnetic stimulation (TMS) therapy has few side effects and comparable therapeutic effects to antidepressant treatment, but few studies have introduced TMS therapy as an initial treatment for MDD. The objective of this study was to retrospectively compare the clinical outcomes between 50 MDD patients without antidepressants (i.e., TMS monotherapy) and 50 MDD patients with antidepressants plus TMS therapy, matched for age, sex, and depression severity. The presence or absence of antidepressant therapy in first-line treatment was determined via a detailed interview by psychiatrists. The study design was a retrospective observational case–control study using the TMS registry data. The key inclusion criteria were adult patients who met the diagnosis of MDD and received 20–30 sessions of intermittent theta-burst stimulation (iTBS) therapy to the left dorsolateral prefrontal cortex (DLPFC). In this study, the Montgomery–Åsberg Depression Rating Scale (MADRS) was used as the primary outcome measure. No significant group differences existed in the baseline MADRS total score between the unmedicated and medicated patient groups. Following TMS therapy, no significant group differences in response rate, remission rate, or relative total score change in the MADRS were observed. The main limitations were the retrospective design and the use of registry data as a source. Our findings suggest that TMS monotherapy may be as effective as TMS add-on therapy to antidepressants when used as the first-line therapy for MDD, but randomized controlled trials are needed.

## 1. Introduction

Major depressive disorder (MDD) is a common mental disorder with a significant prevalence and burden worldwide [1,2]. Globally, an estimated 5% of adults suffer from MDD [3]. In addition, according to the Global Burden of Diseases, Injuries, and Risk Factors Study 2019, depressive disorders were among the top 25 leading causes of global burden in 2019 [4]. Furthermore, the COVID-19 pandemic has exacerbated the prevalence and burden of MDD [5]. In fact, it was estimated that the pandemic added 53 million people with MDD worldwide in 2020 [4]. To make matters worse, MDD is the greatest risk factor for suicide [3]. Currently, psychotherapy and second-generation antidepressants are generally effective treatments for MDD [6], but there are a substantial number of cases in which depressive symptoms do not improve with existing treatments (i.e., treatment-resistant depression (TRD)) [7,8,9]. This reality underscores the importance of recognizing MDD and the urgent need for effective treatments. Continued research and development in this area is crucial to improve the lives of those affected by this disorder.

Antidepressants are commonly used as the first-line treatment for MDD to manage the symptoms and improve the quality of life. However, antidepressants have certain limitations and side effects that can make them problematic as a first-line treatment for some patients with MDD [10,11,12,13,14,15,16,17,18,19]. Antidepressants can cause potential side effects such as weight gain, sleep disturbances, and sexual dysfunction [10,13,14,15,19,20]. These problems are usually temporary or mild but can be bothersome for some patients. Furthermore, not all antidepressants work for all patients. It is common for patients to try multiple medications before finding the right one for them. Consequently, these side effects and delayed onset reduce adherence in patients with depression and are a major impediment to an effective antidepressant response [21]. Moreover, abrupt discontinuation of medications can lead to withdrawal symptoms [22]. Given these limitations, alternative treatment options such as transcranial magnetic stimulation (TMS) are being considered [23,24]. TMS is a noninvasive, non-systemic treatment that uses magnetic fields to stimulate mood-regulating areas and networks in the brain [25,26]. TMS therapy has been found to be an effective and safe alternative to conventional antidepressants for patients with MDD [27,28]. In a prior study comparing the efficacy of ketamine and repetitive TMS (rTMS) in patients with MDD, both treatments significantly improved the symptoms of depression from pre- to post-treatment [29].

TMS uses magnetic pulses to stimulate specific areas of the brain involved in the regulation of mood [26]. TMS can modulate the activity of neurons and neural circuits in the brain and induce long-lasting changes in synaptic plasticity [30]. TMS can also affect the levels and function of a variety of neurotransmitters, including serotonin, dopamine, and gamma-aminobutyric acid (GABA) [31]. In contrast, antidepressants act on the regulation of neurotransmitters that allow the brain to communicate with the nervous system. Antidepressants can increase the concentrations of certain neurotransmitters, such as serotonin and noradrenaline, in the synaptic cleft by promoting their availability and inhibiting their reuptake and breakdown. Antidepressants can also affect the expression and function of receptors and transporters for neurotransmitters, as well as other molecular pathways involved in neuroplasticity and neurogenesis [32]. The key difference between TMS and antidepressants is that TMS is a non-invasive and non-pharmacological intervention that directly targets the brain regions involved in depression, whereas antidepressants are systemic and pharmacological treatments that indirectly modulate brain chemistry. TMS can be an alternative or adjunctive therapy for patients who do not respond to antidepressants or who cannot tolerate their side effects.

With this background, a portion of patients with depression do not wish to receive antidepressant treatment and, in some cases, avoid it due to intolerance or low tolerance to antidepressants, psychological resistance of patients to the potential side effects of antidepressants, or patients’ preference for other treatment options for depression, resulting in a certain number of cases where they often do not receive general and adequate antidepressant treatment.

On the other hand, in addition to antidepressant treatment, psychotherapy, including cognitive behavioral therapy (CBT), is regarded as an effective treatment for MDD [33,34], and it has been reported that a combination of antidepressants and psychotherapy is clinically the most effective treatment for patients with moderate-level MDD [35,36,37]. However, a limitation regarding CBT is that it has not yet been sufficiently validated for severe cases [38] or adolescent cases [39] of depression. Furthermore, in reality, the number of psychiatrists and clinical psychologists who can provide full-fledged psychotherapy is limited, and there is a limitation on public healthcare coverage, making it difficult to provide CBT within the framework of public medical insurance, at least in Japan. Due to such a lack of professional resources, time, and medical costs in actual clinical settings, the main treatment option for depression in Japan, as in other developed countries, tends to be antidepressant treatment alone.

Thus, in recent years, there has been an increasing need for transcranial magnetic stimulation (TMS) therapy as a minimally invasive therapeutic option with fewer side effects than conventional pharmacotherapy. TMS has been recognized as an evidence-based treatment for pharmacoresistant MDD over the past 15 years [31,40]. One of the most extensive trials on TMS noted that 47 percent of patients with TRD responded positively. Of those, approximately one-third of patients achieved full remission of their depression [41]. Moreover, recently, in a double-blind, controlled trial, known as the Stanford Accelerated Intelligent Neuromodulation Therapy (SAINT or SNT), high-dose transcranial magnetic stimulation administered on an accelerated schedule with an fMRI individualized targeting approach resulted in remission in 79% of participants in the trial with severe depression [42]. In this intensive, individualized TMS therapy, remission typically occurred within a few days, and the effects lasted for several months [42].

The appreciation and application of TMS technology have advanced greatly over the past few years [31]. In particular, theta-burst stimulation (TBS) therapy has substantially reduced the treatment time compared with conventional rTMS therapy. Indeed, this form of rTMS (i.e., TBS) now has evidence indicating that it is non-inferior to standard rTMS therapy and provides significant advantages in administration [43]. Furthermore, the individualized targeting approach with MRI navigation has recently gained attention as a new technology that has the potential to optimize and maximize clinical outcomes for each patient [31]. Recent studies indicated that neuroimaging and related approaches may be able to improve TMS targeting methods and potentially identify those patients most likely to respond to TMS therapy [31]. Although these findings are promising, prospective validation studies with large RCTs are needed to individualize and optimize TMS procedures [31]. New approaches, such as accelerated TMS and advanced targeting methods, require further validation of replication and demonstration of their clinical utility in real-world clinical practice [31].

To date, most TMS clinical studies have been premised on the development of novel therapies to be added onto antidepressants for TRD [44]; thus, no clinical studies have introduced TMS therapy as a first-line treatment prior to the initiation of pharmacotherapy. Accordingly, it is currently unclear how much the effectiveness of TMS therapy for depression differs depending on the use of antidepressant medications. Furthermore, Noda et al. recently analyzed the cost-effectiveness of TMS therapy for depression under the public insurance system in Japan, using antidepressant therapy as a contrast, and showed that if one antidepressant treatment fails to achieve a response, switching to TMS therapy immediately thereafter could be more beneficial, not only from a medical point of view but also from a healthcare economic perspective [45]. As such, to our knowledge, there have been no clinical studies directly comparing the effectiveness of TMS monotherapy and TMS add-on therapy for MDD, and a recent cost-effectiveness analysis of TMS therapy versus antidepressant therapy for TRD suggested that introducing TMS monotherapy as a first-line treatment may be theoretically cost-effective. In this context, it is quite reasonable to preliminarily examine the medical effectiveness of TMS monotherapy versus conventional TMS add-on therapy.

Therefore, we aimed to compare the effectiveness of TMS monotherapy versus TMS adjunctive to antidepressants by analyzing registry data matched for age, sex, and depression severity prior to the initiation of TMS therapy for a group of MDD patients who were not receiving antidepressants and a group of MDD patients who were receiving antidepressants. Based on our clinical experience, we anticipated that TMS monotherapy and combination therapy with a TMS add-on to antidepressants would be equally effective in treating patients with depression of moderate severity.

## 2. Materials and Methods

### 2.1. Study Setting

The data included in this retrospective case–control study were collected between December 2020 and October 2023. Specifically, we extracted and analyzed the data of 100 outpatients with MDD (50 medicated patients and 50 unmedicated patients) who received 20 to 30 sessions of intermittent TBS (iTBS) therapy, as described below, to the left dorsolateral prefrontal cortex (DLPFC). In this case–control study, the TMS treatment (iTBS) itself was the same for both groups, and the treatment effects were compared retrospectively, with the “case” being with antidepressant therapy (i.e., antidepressants plus TMS therapy) and the “control” being without antidepressant therapy (i.e., TMS monotherapy), matched for age, sex, and baseline depression severity. The primary endpoints of the present study were the response rate, remission rate, and relative total score change using the Montgomery–Åsberg Depression Rating Scale (MADRS) before and after TMS treatment. Then, we retrospectively compared the differences in the primary endpoints. Herein, clinical response was defined as cases in which the score improved by 50% or more after acute iTBS treatment compared with baseline prior to the start of treatment. On the other hand, remission was defined as cases in which the MADRS score reached 10 or less with therapeutic intervention.

In all cases of TMS therapy at the clinic, informed consent was given on the basis of a personal contract between the physician in charge and the patient. Furthermore, this TMS registry and retrospective observational analysis study was approved by the ITO Yoyogi Mental Clinic Research Ethics Committee (ID: RKK319) and conducted in compliance with the norms and guidelines of the Ethical Guidelines for Medical and Health Research Involving Human Subjects and the Declaration of Helsinki at Tokyo Yokohama TMS Clinic. In accordance with the study protocol of the TMS registry, informed consent was obtained for prospective data, and opt-out was applied for past data.

### 2.2. Extracted Data

The eligibility criteria for this retrospective case–control study were as follows: patients who (1) were 18 years of age or older; (2) met the Diagnostic and Statistical Manual of Mental Disorders (5th edition) (DSM-5) definition of the diagnosis of MDD with standard psychiatric consultations by certified psychiatrists; (3) had no previous history of convulsive seizures; (4) had no other apparent contraindications to TMS therapy; and (5) had received iTBS treatment between 20 and 30 sessions. In this case–control study, data from 50 unmedicated patients with MDD and 50 medicated patients with MDD (i.e., TRD), matched retrospectively for age and sex, were extracted from TMS registry data accumulated at the Tokyo Yokohama TMS Clinic and used for this analysis. Herein, we stratified sex by binary data, age by 3-year intervals, and MADRS score by 3-point intervals and then matched the two groups retrospectively. In addition, we defined TRD for convenience as those who were using one or more antidepressants for a certain period of time during the depressive episode and who still had a MADRS score of more than 15 points in the present study. In the present study, antidepressant treatment for patients with depression consisted of second-generation antidepressants (SSRIs, SNRIs, and mirtazapine), which are recommended as the first-line treatment in many guidelines for depression. Furthermore, the medicated MDD group was taking antidepressants from prior to the start of TMS therapy until at least the end of the therapy. We also confirmed this condition during the patients’ routine outpatient visits.

### 2.3. TMS Therapy Protocol Used in This Study

The TMS therapy protocol used in the case group data extracted for the present study was intermittent theta-burst stimulation (iTBS) of the left dorsolateral prefrontal cortex (DLPFC), with the stimulation intensity set at a 120% resting motor threshold (RMT) and the number of pulses set at 1200 pulses for 6 min, twice the amount of the original protocol (a total of 20 trains of 50 Hz triplet bursts in a 5 Hz rhythm for 2 s followed by 8 s off were administered, totaling 600 pulses in the protocol), based on previous studies [46,47]. The motor threshold was determined via visual confirmation by trained technicians. Specifically, the resting motor threshold was defined as the stimulus intensity at which the patient’s right fingers flexed slightly one out of two times when the hot spot innervating the right fingers in the left motor cortex was stimulated. All cases in the data used for the analysis of this registry study had received a total of 30 sessions of TMS therapy. The Beam F3 method was used to identify the target site on the left DLPFC in each case [48]. For TMS therapy, a MagPro R30 TMS device (MagVenture Inc., Farum, Denmark) equipped with a Cool-B65 TMS coil or a Cool-B70 TMS coil was used.

### 2.4. Clinical Assessment Measure

Patients with MDD were clinically assessed using the Montgomery–Åsberg Depression Rating Scale (MADRS) before and after a total of 30 sessions of TMS therapy. Note that only data from patients who agreed to participate in the TMS registry study were used in the present study. Response to TMS therapy was defined as a 50% or greater improvement in the MADRS score before and after TMS therapy, and remission was defined as a MADRS score of 10 or less at the end of treatment. Table 1 shows the mean MADRS scores (±SD) at baseline.

### 2.5. Statistical Analysis

Statistical analyses were conducted using SPSS software (IBM Corporation, Armonk, NY, USA; version 28.0) for this study. First, the normality of the clinical data was confirmed using the Shapiro–Wilk test. In addition, group comparisons were conducted using the chi-square test for the categorical data and independent *t*-tests for the continuous data, depending on the attributes of the data. The significance level was set at *p* < 0.05 in this study.

## 3. Results

### 3.1. Clinicodemographic Information

First, in this study, the clinical data of 50 unmedicated patients with MDD (33 males and 17 females; mean age 36.6 ± 11.3 years) and 50 medicated patients with MDD (33 males and 17 females; mean age 38.3 ± 11.9 years) were used and analyzed. At baseline, prior to the start of treatment, there were no significant group differences between the unmedicated MDD group and the medicated MDD group with respect to the number of females, age, and the MADRS score (Table 1).

### 3.2. Clinical Outcomes

Next, the longitudinal changes in the MADRS score between the two groups demonstrated no significant differences in treatment efficacy (Table 2). In addition, no significant differences in response and remission rates in the total scores of the MADRS were observed (Table 2).

### 3.3. Adverse Events and Side Effects

Finally, with respect to the retrospective case–control data included in this study, none of the cases showed any serious adverse events, including convulsive seizures or manic switching. The most common side effect was stimulation site pain associated with TMS therapy in both groups, with seven cases reported in the TMS therapy alone group and eight cases in the TMS therapy plus antidepressant treatment group, with no obvious group differences between the two groups.

## 4. Discussion

In this study, we retrospectively matched age, sex, and severity of depression between the antidepressant-treated and non-treated groups among patients with a diagnosis of MDD using the TMS database that was registered at the Tokyo Yokohama TMS Clinic and posteriorly assessed the effectiveness of TMS therapy for both groups. With the MADRS score as the primary outcome measure in the present study, the effectiveness of iTBS treatment for 50 patients without antidepressants (i.e., iTBS monotherapy) and 50 patients with antidepressants (i.e., iTBS add-on therapy to antidepressants) were not significantly different between the two groups, with no apparent differences in the degree of improvement in depressive symptoms or the frequency of adverse events.

To the best of our knowledge, there have been few clinical studies in which TMS therapy was administered as a first-line treatment for MDD without antidepressant treatment. As has been repeatedly reported [49], the mechanism of action of TMS therapy for MDD, including TRD, is assumed to involve the normalization of excessive activation of the default mode network (DMN) [50], including the subgenual anterior cingulate cortex [51]. Although the pathological basis and pathophysiology of depression are complex and diverse and not yet fully understood, neuroimaging studies have revealed a “depression network” common to depression [52]. Specifically, Siddiqi et al. examined how brain lesions, TMS, and deep brain stimulation affect symptoms of depression by targeting specific brain circuits. Then, they analyzed 14 datasets from different studies and found that the brain regions most associated with depression severity were linked by a common circuit. This circuit involved regions such as the subgenual cingulate, ventromedial prefrontal cortex, and dorsolateral prefrontal cortex, which are known to be involved in depression [53]. Moreover, Siddiqi et al. took a reverse approach and identified brain circuits associated with the improvement of different depressive symptoms following TMS therapy to the left prefrontal cortex. They mapped the stimulation sites in patients receiving TMS therapy to the underlying brain network using functional connectivity magnetic resonance imaging (MRI). Next, they identified circuits that correlated with symptom improvement in two independent patient cohorts. They found that dysphoric symptoms and anxiety/somatic symptoms responded better to the stimulation of distinct circuits. These neural circuits were consistent across datasets and were more specific to active stimulation compared with sham stimulation [54]. Furthermore, Siddiqi et al. found that the most effective TMS and deep brain stimulation targets were functionally connected to the subgenual cingulate cortex and that lesions in the same regions caused depression. These findings suggest that causal circuit mapping can identify common neural pathways that underlie different neuromodulation modalities and may guide the discovery of new treatment targets for neuropsychiatric disorders including depression [55].

Thus, ultimately, if the network can be normalized, any approach, whether pharmacotherapy or neuromodulation, may have a therapeutic effect. However, since the appropriate therapeutic approach differs from patient to patient, it is necessary in the future to examine, develop, and flexibly apply a stratified treatment to some extent according to the pathophysiology of the patient with depression, instead of proceeding with a homogeneous and uniform treatment in a one-size-fits-all approach [56].

Prior research has shown that patients with fewer failed antidepressant trials (i.e., less treatment-resistant patients) are more likely to respond to TMS therapy [57] and are consequently more medically cost-effective [45]. In the case of MDD, if appropriate treatment is not implemented in the early stage, depression may develop into chronic and refractory depression, resulting in the risk of transitioning to treatment-resistant or difficult-to-treat depression [58]. To date, no clinical studies have directly compared the effectiveness between TMS therapy for medication-naïve/free patients with depression (i.e., TMS monotherapy) and medication-resistant depression (i.e., antidepressants plus TMS add-on therapy for TRD). Nevertheless, previous studies have suggested that TMS add-on therapy may lead to better therapeutic outcomes compared with TMS monotherapy [27,37,59,60]. Specifically, Baeken and colleagues demonstrated that typical antidepressant monotherapy for depression results in a response of close to 50% and remission of more than about 35%, while TMS monotherapy leads to a response of close to 60% and remission of more than about 35% [37]. Therefore, a response rate of 80% and a remission rate of 64% with TMS monotherapy for MDD in our TMS registry data analysis is numerically better than the results of a previous observational study. To directly address this clinical question, RCTs in both treatment arms are needed; however, it is also even more crucial to develop individualized and optimized TMS treatment protocols from a different perspective [45].

In addition, a certain number of patients with MDD have a low tolerance to pharmacotherapy and are unable to receive adequate doses of antidepressant therapy because of the side effects that come to the fore with usual antidepressant treatment [61]. Even though our results are preliminary, the use of TMS monotherapy as a first-line treatment for MDD would be clinically beneficial for patients with depression, specifically, those who cannot take antidepressants because of severe hepatic, renal, or cardiac dysfunction, women who are pregnant or planning to become pregnant, postpartum patients with depression who are breastfeeding, and the elderly who suffer from severe adverse side effects from antidepressants. Moreover, apart from low tolerance, there is a portion of patients who originally have a strong psychological resistance to pharmacotherapy [62,63]. In such cases, it may be worthwhile to consider TMS therapy with fewer side effects as a first-line treatment based on shared decision making between the patient and the physician, without necessarily prioritizing antidepressant therapy, which is the first-line treatment in most current algorithms and guidelines for the treatment of depression. This may even result in a better prognosis for some patients with depression. In the clinical practice of depression in reality, a certain number of cases exist in which patients with MDD do not receive adequate doses and a sufficient duration of antidepressant medication due to concerns about side effects, resulting in a lack of therapeutic response and a poor prognosis. The results of this real-world retrospective TMS registry data analysis show that no clear differences were observed in terms of treatment outcomes between the group of cases in which TMS therapy was introduced initially without first-line pharmacotherapy for MDD (i.e., iTBS therapy for unmedicated MDD) and the group of cases in which TMS therapy was introduced as a second-line treatment because the patients developed treatment resistance after receiving conventional pharmacotherapy (i.e., iTBS therapy for TRD). Thus, from a clinical perspective, the results of this study suggest that an approach that considers and introduces TMS therapy for MDD patients from the beginning may also be worthwhile. In fact, a recent study conducted in Japan that analyzed in detail the cost-effectiveness of TMS therapy in contrast with antidepressant treatment under public medical insurance for TRD also suggests that early introduction of TMS therapy after diagnosis of MDD could be beneficial [45].

This study has some limitations. First, the present study was a retrospective case–control study using registry data as a source. Therefore, future rigorous validation with a prospective RCT is needed to rigorously confirm the results of the present study. Second, because the present study retrospectively compared the combination therapy with antidepressants and the TMS therapy group, which showed medication resistance, to the TMS monotherapy group, which mainly showed low tolerance to medication, the presence of medication resistance may have been a potential confounding factor for the treatment effects of the two groups. Although this was unavoidable for the objective and design of the present study, it would be necessary to consider controlling for treatment resistance as a covariate by indexing it in the future. Third, because the targeting method for the stimulation sites in the data used in the present study was the Beam F3 method rather than the method using an MRI-guided navigation system, the stimulation sites on the left DLPFC may not be rigorously accurate. However, this was an unavoidable limitation because the present study used clinical data in a real-world setting.

## 5. Conclusions

This present study demonstrated that iTBS monotherapy may be as effective as iTBS add-on therapy to antidepressants for patients with MDD, even when patients initially receive TMS therapy without taking antidepressants due to concerns about side effects from pharmacotherapy. Since such patients exist in a certain number, further validation via rigorous RCTs is warranted to confirm this finding in the future. Again, this retrospective analysis study utilizing TMS registry data suggests that TMS monotherapy may be an effective first-line treatment for some MDD patients, but further RCTs are needed to confirm its efficacy compared with antidepressant monotherapy as well as combination therapy with antidepressants and TMS add-on therapy. Furthermore, if the present findings are confirmed in future RCTs, it may be necessary to revise treatment guidelines for MDD to include TMS monotherapy as a first-line option in selected patients. Finally, it is important to note, however, that on an individual level, the effectiveness of TMS monotherapy will depend on a variety of factors, including the individual’s specific condition, severity of depression, and responsiveness to previous treatment. Therefore, it is of utmost importance to consult with psychiatrists who specialize in TMS therapy to determine the best treatment approach.

## Figures and Tables

**Table 1:** Clinicodemographic information at baseline of patients with MDD without and with antidepressants.

	MDD without Antidepressants	MDD with Antidepressants	Statistical Results of Group Comparisons (95% CI)
*n*	50	50	-
Males	33	33	-
Females	17	17	-
Age (mean ± SD) (years)	36.6 (±11.3)	38.3 (±11.9)	t_98_ = 0.74; *p* = 0.46 (−6.3 ≤ 95% CI ≤ 2.9)
MADRS score at baseline	30.2 (±4.9)	28.6 (±6.0)	t_98_ = 1.44; *p* = 0.15 (−0.60 ≤ 95% CI ≤ 3.8)

MDD: major depressive disorder: CI: confidence interval; SD: standard deviation; MADRS: Montgomery–Åsberg Depression Rating Scale.

**Table 2:** Therapeutic efficacy in patients with MDD without antidepressants (iTBS monotherapy) and with antidepressants (iTBS add-on therapy to antidepressants) assessed with the MADRS score.

	MDD without Antidepressants	MDD with Antidepressants	Statistical Results of Group Comparisons (95% CI)
Percentage changes in MADRS score (%)	67.5 (±21.3)	61.6 (±25.9)	t_98_ = 1.25; *p* = 0.22 (−3.5 ≤ 95% CI ≤ 15.3)
Response rate with MADRS score (response/non-response)	80% (*n* = 40/*n* = 10)	74% (*n* = 37/*n* = 13)	χ^2^ (1) = 0.51; *p* = 0.48 OR: 1.4 (0.28 ≤ 95% CI ≤ 1.8)
Remission rate with MADRS score (remission/non-remission)	64% (*n* = 32/*n* = 18)	58% (*n* = 29/*n* = 21)	χ^2^ (1) = 0.38; *p* = 0.54 OR: 1.3 (0.35 ≤ 95% CI ≤ 1.7)

MDD: major depressive disorder: CI: confidence interval; OR: odds ratio; MADRS: Montgomery–Åsberg Depression Rating Scale.

## Data Availability

The data analyzed in the present study will be available from the corresponding author upon reasonable request.
